# Cloning, expression and characterization of an ethanol tolerant GH3 β-glucosidase from *Myceliophthora thermophila*

**DOI:** 10.7717/peerj.46

**Published:** 2013-02-26

**Authors:** Anthi Karnaouri, Evangelos Topakas, Thomas Paschos, Ioanna Taouki, Paul Christakopoulos

**Affiliations:** 1 Biotechnology Laboratory, School of Chemical Engineering, National Technical University of Athens, Athens, Greece; 2 Biochemical and Chemical Process Engineering, Division of Sustainable Process Engineering, Department of Civil, Environmental and Natural Resources Engineering, Luleå University of Technology, Luleå, Sweden

**Keywords:** Glycoside hydrolase family 3, Myceliophthora thermophila, *Pichia pastoris*, Overexpression, β-glucosidase, Transglycosylation, Ethanol tolerance, Thermophilic

## Abstract

The β-glucosidase gene *bgl3a* from *Myceliophthora thermophila*, member of the fungal glycosyl hydrolase (GH) family 3, was cloned and expressed in *Pichia pastoris*. The mature β-glucosidase gene, which results after the excision of one intron and the secreting signal peptide, was placed under the control of the strong alcohol oxidase promoter (*AOX1*) in the plasmid pPICZαC. The recombinant enzyme (90 kDa) was purified and characterized in order to evaluate its biotechnological potential. Recombinant *P. pastoris* efficiently secreted β-glucosidase into the medium and produced high level of enzymatic activity (41 U/ml) after 192 h of growth, under methanol induction. *Mt*Bgl3a was able to hydrolyze low molecular weight substrates and polysaccharides containing β-glucosidic residues. The *K*_*m*_ was found to be 0.39 mM on p-β-NPG and 2.64 mM on cellobiose. Optimal pH and temperature for the p-β-NPG hydrolysis were 5.0 and 70 °C. The β-glucosidase exhibits a half life of 143 min at 60 °C. Kinetic parameters of inhibition were determined for D-glucose, D-xylose and D-gluconic acid, indicating tolerance of the enzyme for these sugars and oxidized products. The recombinant enzyme was stimulated by short chain alcohols and has been shown to efficiently synthesize methyl-D-glucoside in the presence of methanol due to its transglycosylation activity. The stability of *Mt*Bgl3a in ethanol was prominent, and it retained most of its original activity after we exposed it to 50% ethanol for 6 h. The high catalytic performance, good thermal stability and tolerance to elevated concentrations of ethanol, D-xylose and D-glucose qualify this enzyme for use in the hydrolysis of lignocellulosic biomass for biofuel production, as part of an efficient complete multi-enzyme cocktail.

## Introduction

Cellulose that is the most abundant biopolymer on earth, is formed by β-(1,4)-linked D-glucose units, where adjacent D-glucoses are flipped making cellobiose the fundamental repeating unit (Mansfield, Mooney & Saddler, 1999). Polymers of cellulose form robust microfiber structures, which are stabilized by inter- and intramolecular hydrogen bonds and van der Waals interactions between D-glucose residues contributing to its recalcitrance (Cheng et al., 2011). Although cellulose is mainly present as crystalline fibers that are highly resistant to hydrolysis, its content in biomass is typically larger compared to hemicellulose, and as a result, cellulases are the key enzymes for bioethanol production (Ragauskas et al., 2006). Cellulases have been widely applied in industry in different sectors such as in the textile industry for cotton softening and denim finishing, in the detergent market for colour care, cleaning, and anti-deposition, in the food industry for mashing and in the pulp and paper industries for de-inking, improvement, and fiber modification (Bhat, 2000). Enzymes that modify complex carbohydrates, such as cellulases, together with their accessory noncatalytic carbohydrate binding modules (CBMs), have been grouped into sequence-based families on the continuously updated Carbohydrate-Active EnZymes (CAZy) database (http://www.cazy.org/) (Cantarel et al., 2009). β-Glucosidases (EC 3.2.1.21) which act on soluble cello-oligosaccharides produced by the action of β-1,4-endoglucanases (EC 3.2.1.4) and cellobiohydrolases (EC 3.2.1.91), including cellobiose, towards the release of D-glucose, belong to GH families 1, 3, 5, 9, 30 and 116. β-Glucosidases hydrolyze soluble cellodextrins and cellobiose to D-glucose and thus relieve the system from end product inhibition (Himmel et al., 2007). As shown in various studies (Bezerra & Dias, 2005; Gruno et al., 2004; Zhao et al., 2004) the cellobiose released during the hydrolysis of lignocellulose for ethanol production has a high inhibitory effect on cellulases. Thus the need of instant cellobiose removal is crucial for the efficiency of the process. Due to the fact that the most common cellulolytic commercial complex (Celluclast 1.5L) lacks β-glucosidase activity, efforts have been made for the production of an enzyme supplement that will be rich in this enzymatic activity (Zhang et al., 2010; Gurgu et al., 2011; Pitson, Seviour & McDougall, 1997). The presence of sufficient β-glucosidase activity in the enzyme mixture is shown to increase the hydrolysis performance by more than 20% reaching even 40% increase in the total ethanol production of the processes (Xin, Yinbo & Peiji, 1993; Han & Chen, 2008). Here lies the need of β-glucosidase activity in the lignocellulosic multi-enzyme systems.

Recently, β-glucosidases have become the focus of many applied studies because they are essential part not only in the cellulose breakdown but also in the synthesis of oligomers and other complex molecules, such as alkyl-glucosides. The synthetic behavior is a result of the transglycosylation activity that has occurred by the presence of a stronger nucleophile compared to water, such as methanol. Therefore, these enzymes can be used for synthesizing a variety of glycoconjugates, such as alkyl glucosides, aminoglycosides and special disaccharide fragments of phytoalexin-elecitor oligosaccharides, which are involved in plant and other microbial defence mechanisms (Bhatia, Mishra & Bisaria, 2002). The enzymatic production of alkyl glycosides, which are surfactants with good biodegradability and low toxicity, is attractive forming stereochemically well-defined products. To date, much of the effort in the enzymatic synthesis of alkyl glucosides has been placed into GH1 enzymes (Hansson & Adlercreutz, 2001; Goyal, Selvakumar & Hayashi, 2001), however, there is still a great need to find better glycosidases to compete traditional chemical synthesis (Goyal, Selvakumar & Hayashi, 2001; Turner et al., 2006).

The thermophilic fungi *Myceliophthora thermophila* (synonym *Sporotrichum thermophile*) ATCC 42464, is an exceptionally powerful cellulolytic organism which synthesizes a complete set of enzymes necessary for the breakdown of cellulose. This ability of *M. thermophila* in combination with the recently published genome sequence (Genome Portal, Join Genome Institute, University of California; http://genome.jgi-psf.org; (Berka et al., 2011)) has raised interest for discovering novel cellulolytic enzymes with thermophilic properties. This paper describes, for the first time, the successful cloning of the complete genomic DNA sequence of *M. thermophila* β-glucosidase gene belonging to GH3 family, and its heterologous expression in methylotrophic yeast *P. pastoris*. The enzyme hydrolyses glucosidic substrates with high specific activity, is stimulated by alcohols and has been shown to be an efficient biocatalyst in alkyl glucoside synthesis at increased cellobiose concentrations due to its transglycosylation activity. The enzyme exhibited a combined tolerance to ethanol, pH and temperature, which offers a good candidate to be used in SSF processes for biofuel production. These characteristics reflect potential commercial significance of the enzyme.

## Materials and Methods

### Enzymes and chemicals

KOD Hot Start^®^ DNA polymerase was purchased from Novagen (USA), while restriction enzymes were purchased from TAKARA (Japan). GeneJET™ Plasmid Miniprep and Nucleospin Gel Clean up kits were purchased by Fermentas (USA) and Macherey Nagel (Germany), respectively. All other chemicals were of analytical grade. The substrates lichenan, cellobiose, CMC, laminarin (*Laminaria digitata*), 4-methylumbelliferyl β-D-glucopyranoside (MeUmbGlc), methyl-β-D-glucopyranoside, *p*-NPh-β-D-glucopyranoside (*p*-β-NPG), *p*-NPh-α-D-glucopyranoside (*p*-α-NPG), *p*-NPh-β-D-glactopyranoside (*p*-β-NPGal), *p*-NPh-α-D-galactopyranoside (*p*-α-NPGal), *p*-NPh-β-D-cellobioside (*p*-NPCell), were obtained from Sigma-Aldrich, (St. Louis, MO). Barley β-glucan and wheat arabinoxylan were purchased from Megazyme. Crystalline cellulose Avicel and birchwood xylan were from Merck (Darmstadt, Germany).

### Cloning of *bgl3a* and transformation of *P. pastoris*

For the cloning of the β-glucosidase gene from *M. thermophila*, *Escherichia coli* One Shot^®^ Top10 (Invitrogen, USA) and Zero Blunt^®^ PCR Cloning Kit (Invitrogen, USA) were used as the host-vector system. *P. pastoris* host strain X-33 and pPICZαC (Invitrogen, USA) were used for protein expression. The WT strain of *M. thermophila* ATCC 42464 was maintained on 1.5% malt-peptone-agar slants at 4 °C. *P. pastoris* was routinely grown in shaking flasks at 30 °C according to the instructions in the EasySelect™ *Pichia* Expression Kit (Invitrogen, USA). Genomic DNA was prepared and isolated as previously described (Topakas et al., 2012).

An *E. coli*/*P. pastoris* vector, pPICZαC, was used to achieve secreted expression of *Mt*bgl3a. pPICZαC contains the tightly regulated *AOX1* promoter and the *Saccharomyces cerevisiae* α-factor secretion signal located immediately upstream of the multiple cloning site (Higgins et al., 1998). The gene coding for the hypothetical protein *Mt*Bgl3a (Model ID 66804; chromosome_3:4861135-4863642) was PCR amplified from genomic DNA using primers EF/ER (Table 1) designed accordingly to the available gene sequence (http://genome.jgi-psf.org/, DOE Joint Genome Institute, (Berka et al., 2011) including the *ClaI* and *XbaI* restriction enzyme sites at their respective 5’-ends. A high fidelity KOD Hot Start^®^ DNA polymerase producing blunt ends was used for the DNA amplification, which was carried out with 30 cycles of denaturation (20 s at 95 °C), annealing (10 s at 60 °C), and extension (50 s at 70 °C), followed by 1 min of further extension at 70 °C. In order to determine the DNA sequence, the PCR product was cloned into the pCRBlunt^®^ vector according to the method described by the Zero Blunt^®^ PCR Cloning Kit.

Intron removal was achieved using the molecular technique of overlap extension polymerase chain reaction (OEPCR) (Topakas et al., 2012) using the polymerase KOD Hot Start^®^ (Novagen, USA). Two complementary DNA primers per intron, two external primers (EF/EeR, EeF/ER, Table 1) and the appropriate PCR amplification process were used to generate two DNA fragments harbouring overlapping ends. The recombinant plasmid pCRBlunt/*bgl3a*, at an appropriate dilution, was used as template DNA and the PCR conditions for each reaction are given as the following: 95 °C for 2 min, ensued by 30 cycles of 95 °C for 20 s, 60 °C for 10 s and 70 °C for 16 s (fragment 407 bp) or 20 s (fragment 1795 bp) respectively, with a final extension step at 70 °C for 1 min. The two PCR products were combined together in a subsequent hybridization reaction. The generated “fusion” fragment was amplified further by overlapping PCR through the utilization of the two external primers, EF end ER, with an initial denaturation step at 95 °C for 2 min, followed by 45 cycles at 95 °C for 20 s, 60 °C for 10 s, 70 °C for 25 s and a final extension step at 70 °C for 1 min. An extended annealing was performed (25 min) in order to improve base-pairing between the complementary ends of each fragments that have to be fused. The produced *bgl3a* DNA was digested with the enzymes *ClaI* and *XbaI* and the DNA fragment gel-purified before cloning into the pPICZαC vector, resulting in the recombinant pPICZαC/*bgl3a* which was amplified in *E. coli* TOP10F′, and the transformants were selected by scoring for Zeocin™ resistance (25 µg/ml). The recombinant vector pPICZαC/*bgl3a* was confirmed by restriction analysis and DNA sequencing and finally transformed into *P. pastoris*.

**Table 1:** PCR primers for amplification and intron splicing.

Primer	Primer sequences
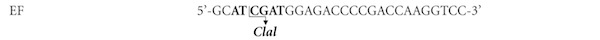	
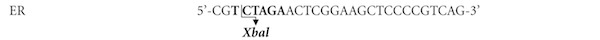	
EeR	5’-GTGAAGTTGGTGTATGACAGCCCAAACCCAAACTCGTACC-3’
EeF	5’-CATACACCAACTTCACGTACGCC-3’

The recombinant plasmid pPICZαC/*bgl3a* was linearized with *SacI*, and then transformation of *P. pastoris* and cultivation in shaken flasks were performed according to the EasySelect™ *Pichia* Expression Kit. High-level expression transformants were screened from the YPDS plates containing Zeocin™ at a final concentration of 100 µg/ml. The presence of the *bgl3a* gene in the transformants was confirmed by PCR using yeast genomic DNA as template and gene specific primers (EF and ER; Table 1).

### Screening of recombinant *P. pastoris* transformants and production of the recombinant protein

To screen the *P. pastoris* transformants for β-glucosidase expression, 50 colonies were plated out on MM (1.34% (w/v) yeast nitrogen base, 4 × 10^−5^% (w/v) biotin and 0.5% (v/v) MeOH top agar at a density of 1 colony/cm^2^. After incubation at 30 °C for 24 h, the plates were overlaid with 4 ml of 1% agarose containing 10 mM MeUmbGlc and incubated at room temperature for up to 5 min. The plates were inspected regularly under UV light for fluorescent haloes surrounding recombinant colonies, which is indicative of MeUmbGlc hydrolysis.

For the quantification of β-glucosidase activity found in the fluorescent positive *P. pastoris* colonies, the different transformants were cultivated in BMGY medium for 18–24 h, at 30 °C in a shaker (200 rpm) and then inoculated into the production medium BMMY reaching OD_600_ = 1. The extracellular secreted protein was tested for β-glucosidase activity after 24 h of incubation at 30 °C and 200 rpm.

The best recombinant *P. pastoris* harbouring *bgl3a* gene was grown and harvested, as previously described (Topakas et al., 2012). The cultures were kept in a shaking incubator at 30 °C for 6 days (200 rpm) with the addition of 0.75 ml methanol once a day to maintain induction (0.5% v/v).

### Purification of recombinant *Mt*bgl3a

The recombinant *P. pastoris* harbouring *bgl3a* gene was grown and harvested, as previously described (Topakas et al., 2012). The cultures were kept in a shaking incubator at 30 °C for 6 days (200 rpm) with the addition of 0.75 ml methanol once a day to maintain induction (0.5% v/v).

For the purification of the recombinant β-glucosidase, 800 ml of culture broth was centrifuged and concentrated 30-fold using an Amicon ultrafiltration apparatus (Amicon chamber 8400 with membrane Diaflo PM-30, exclusion size 30 kDa), (Millipore, Billerica, USA). The concentrate was dialyzed overnight at 4 °C against a 20 mM Tris-HCl buffer containing 300 mM NaCl (pH 8.0) and loaded onto a immobilized metal-ion affinity chromatography (IMAC) column (Talon, Clontech; 1.0 cm i.d., 15 cm length) equilibrated with the same buffer. The column was first washed with 300 ml buffer, then a linear gradient from 0 to 100 mM imidazole in 20 mM Tris-HCl buffer containing 300 mM NaCl (60 ml, pH 8.0) was applied at a flow rate of 2 ml/min. Fractions (2 ml) containing β-glucosidase activity were concentrated and the homogeneity was checked by sodium dodecyl sulphate-polyacrylamide gel electrophoresis (SDS-PAGE) using 10% acrylamide separating gels. For the determination of isoelectric point (pI), isoelectric focusing (IEF) was performed with the Phastsystem using PhastGel IEF (Amersham Biosciences AB) using broad-range IEF markers (pH 3–9) from Pharmacia. Both gels were stained with Coomassie brilliant blue G-250.

### Enzyme characterization

The β-glucosidase activity was determined by incubating the enzyme with *p*-β-NPG. The enzymatic reaction mixtures (1 ml) containing 50 µl of enzyme solution and 1 mM *p*-β-NPG (final concentration) in 0.1 M citrate-phosphate buffer pH 5.0 were incubated for 10 min at 50 °C. The amount of *p*-nitrophenol (*p* NP) released was measured at A_410_, after addition of 0.2 ml 1 M Na_2_CO_3_ to the reaction mixtures, using a standard curved prepared under the same conditions.

The optimal temperature was determined using the standard assay procedure at temperatures ranging from 30 to 80 °C in 0.1 M citrate-phosphate buffer pH 5.0. Temperature stability was determined by measuring the residual activity under the standard assay procedure, after incubation of purified *Mt*bgl3a at various temperatures for different amount of time in 50 mM MOPS buffer (pH = 6.5) in the presence of 1 mg/ml BSA. The optimal pH was determined by the standard assay at 50 °C over the pH range 3.0–11.0 using either 0.1 M citrate-phosphate buffer pH 3.0–7.0, 0.1 M Tris-HCl pH 7–9 or 0.1 M glycine-NaOH buffer pH 9–11. The stability at different pH was determined after incubating the enzyme in the above buffers at 4 °C for 4 h and then measuring the activity remaining using the standard assay.

The substrate specificity of *Mt*bgl3a against *p*-α-NPG, *p*-β-NPGal, *p*-α-NPGal was tested with reaction mixtures containing 0.064 mg *Mt*bgl3a and 5 mM of each substrate under the standard assay conditions. Enzyme activity on *p*-NPCell was tested at the same conditions, using 1 mM substrate. Enzyme activity on multiple polysaccharide substrates (lichenan, barley β-glucan, laminarin, Avicel) or birchwood xylan was also investigated. Enzyme activity was determined after incubation in 0.1 M citrate-phosphate buffer (pH 5.0) containing 1.0% of each substrate at 50 °C for 15 min. The amount of reducing sugars released was estimated using the dinitrosalicylic acid reagent (DNS) (Miller, 1959), using D-glucose for the standard curve. The activity on cellobiose was estimated by assaying the amount of released D-glucose using GOD–POD method (Lin, Pillay & Singh, 1999). One unit of activity was defined as the amount of enzyme which released 1 µmol of D-glucose equivalents or *p*-nitrophenol (*p* NP) per min under assay conditions. The protein was determined by the absorbance at 280 nm using molar extinction coefficient of 115655 M^−1^ cm^−1^ (Stoscheck, 1990).

The values of the Michaelis constant (*K*_*m*_) and the maximum velocity (*V*_max_) for *Mt*bgl3a were determined by incubating the enzyme in 100 mM citrate-phosphate buffer pH 5.0 at 40 °C with *p*-β-NPG and cellobiose at concentrations ranging from 0.1 to 10 mM. The inhibition of *Mt*bgl3a by D-glucose and xylose was determined by assay the enzymatic activity on *p*-β-NPG in the presence of different inhibitor concentrations. Data were fitted to the Michaelis–Menten equation to generate estimates of values for *K*_*m*_, *V*_max_ and *K*_*i*_, using GraFit data analysis software that also gives an estimate of the standard error of each parameter (Leatherbarrow, 1998).

The effect of D-gluconic acid produced by oxidation of D-glucose on the activity of *Mt*bgl3a was examined. Chemical conversion of D-glucose to aldonic acid/lactone took place through a mild oxidation method that has been shown to selectively oxidize the hemiacetal carbon (C1) of carbohydrates to generate aldonic acids (Forsberg et al., 2011). The inhibition of *Mt*bgl3a by D-gluconic acid was determined by assay the enzymatic activity on *p*-β-NPG, as described above.

The effects of various metal ions or other substances at 10 mM on *Mt*bgl3a activity were determined by preincubating the enzyme with the individual compounds in 100 mM citrate-phosphate buffer pH 5.0 at 4 °C for 40 min. Activities were then measured at 50 °C, under standard assay conditions, in the presence of the metal ions or chemical agents. The activity assayed in the absence of metal ions or agents was recorded as 100%.

The effect of alcohols (ethanol, methanol and propanol) as strong nucleophile reagents on the hydrolysis of *p*-β-NPG was studied. Reaction mixtures containing 1 mM *p*-β-NPG in 100 mM citrate phosphate buffer, pH 5.0, with varying concentrations of short chain alcohols were incubated at 50 °C and the activity was measured under standard assay conditions. The stability at various concentrations of ethanol up to 50% (v/v) at 30 °C was determined, after incubating the enzyme in 100 mM citrate-phosphate buffer pH 5.0 for 6 h and then measuring the residual activity using the standard assay. Transglycosylation activity was examined using cellobiose as a donor and methanol as an acceptor. A 2 ml incubation mixture contained 20% (v/v) of methanol, 0.02% NaN_3_ and different concentrations of cellobiose (1, 2, 4, 5 and 6% w/v) in 0.1 M citrate-phosphate buffer, pH 5.0 and 64 µg mg of enzyme was used. The reaction mixtures were incubated at 50 °C and samples were withdrawn at different time intervals for 5 h. Methyl-D-glucoside synthesis was monitored by employing an HPLC system (Shimadzu LC-20AD) equipped with a refractive index detector (Shimadzu RID 10A) and a Macherey-Nagel CC 250 × 4.6 mm Nucleosil 100-5 NH2 column. The mobile phase was acetonitrile:water (87:13 v/v) and the sugars were eluted at a flow rate of 1 ml/min, as described previously (Tsitsimpikou et al., 1997). The products were quantified based on peak areas using standard methyl-β-D-glucopyranoside, D-glucose and cellobiose.

## Results and Discussion

### Identification, cloning and expression of *Mt*bgl3a

*M. thermophila* genome, together with *Thielavia terrestris*, are the first described for thermophilic eukaryotes and the first complete telomere-to-telomere genomes for filamentous fungi (Berka et al., 2011). *M. thermophila* is well known for its ability in hydrolyzing all major polysaccharides found in biomass, as demonstrated recently in genomic analyses and experimental data obtained the last two decades. The translation of *bgl3a* open reading frame (ORF) (Model ID 66804) from the *M. thermophila* genome database shows significant primary sequence identity with known β-glucosidases which have been classified to family GH3 on CAZy database (http://www.cazy.org/; Cantarel et al., 2009).

The putative β-glucosidase shows high sequence identity with β-glucosidases identified in *Trichoderma* species, such as BGL1 (70%) from *Trichoderma viride* (Liu et al., 2004) and BGL1 (71%) from *Hypocrea jecorina* (anamorph *T. reesei*) (Mach, 1993). The hypothetical protein of 66804 was selected as a candidate β-glucosidase and the corresponding gene, which was provisionally named *bgl3a*, was cloned and used to transform *P. pastoris* X-33; the encoded enzyme named *Mt*Bgl3a was expressed and finally characterized.

After selection of *P. pastoris* transformants by their ability to produce fluorescent haloes under UV light when covered with agar containing MeUmbGlc substrate, ten colonies Zeocin™ resistant were screened for protein expression and secretion under methanol induction. All transformants produced a major secreted protein product of ca. 90 kDa upon examination of culture supernatants by SDS-PAGE, whereas no protein could be detected with the vector control (data not shown).

**Figure 1:**
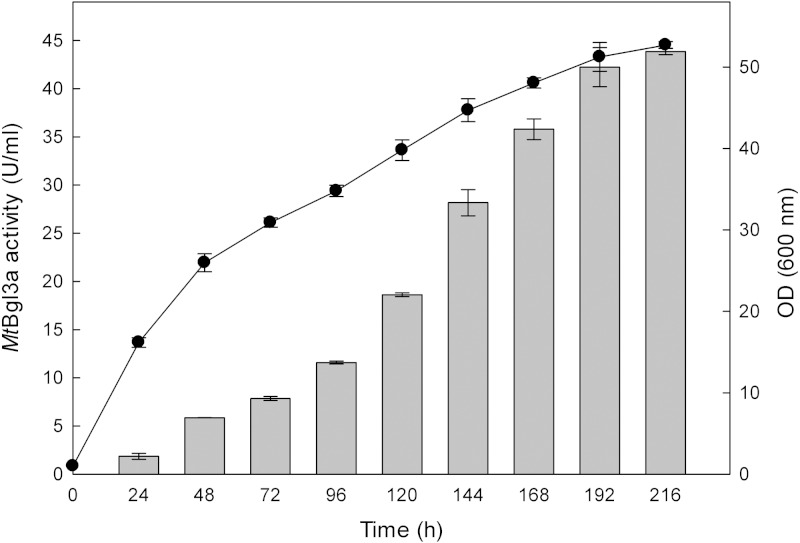
Time course of *Mt*Bgl3a activity (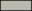) and biomass (●) production of the recombinant *P. pastoris* harbouring the *bgl3a* gene. The β-glucosidase was expressed in culture broth by induction with 0.5% methanol and measured with p-β-NPG as substrate.

The production of β-glucosidase activity by the transformants was confirmed using *p*-β-NPG, as detailed in the Materials and Methods section, and the clone showing the highest activity was retained for further study. β-Glucosidase activity could be first detected in the medium 24 h after inoculation and peaked at 192 h with a titer of 41 U/ml (Fig. 1). The *P. pastoris* expression system has been used successfully to study recombinant glucosidases from various fungi species, like for example from *Paecilomyces thermophila* (PtBglu3) (Yan et al., 2012) and *Aspergillus fumigatus* (nBgl3) (Liu et al., 2012). The methylotrophic yeast *P. pastoris* expression system, as a eukaryotic expression system, has been a favorite system for expressing heterologous proteins due to its many advantages, such as protein processing, protein folding and post-translational modification (Cereghino & Cregg, 2004).

### Protein analysis of *Mt*Bgl3a

The ORF of *bgl3a* encodes a protein of 733 amino acids including a secretion signal peptide of 17 amino acids MTLQAFALLAAAALVRG based upon the prediction using SignalP v4.0, which is a web-based program (http://www.cbs.dtu.dk/services/SignalP/). The predicted mass and isoelectric point (pI) of the mature protein was 79819 Da and pH 5.05, respectively, by calculations using the ProtParam tool of ExPASY (http://web.expasy.org/protparam/).

**Figure 2:**
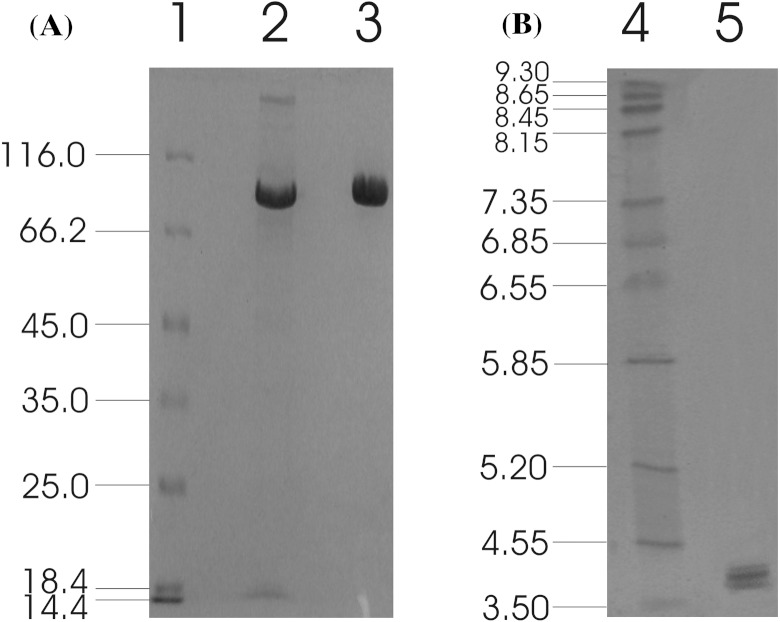
SDS-PAGE (A) and IEF (B) of *Mt*Bgl3a. (A) Lanes: 1, LMW standard protein markers; 2, *P. pastoris* culture broth; 3, purified *Mt*Bgl3a. (B) Lanes: 1, standard protein markers with pI range 3.5–9.3; 2, purified *Mt*Bgl3a.

The recombinant enzyme was purified from the concentrated culture broth by IMAC, with a final concentration of 1.28 mg/ml. The homogeneity of the purified recombinant *Mt*Bgl3a was examined on a SDS-PAGE, which appeared as a single band. The molecular weight was estimated to be ca. 90 kDa (Fig. 2A), which appears to be higher than the predicted value using the ProtParam tool of ExPASY (79819 Da) considering the presence of the myc epitope and the polyhistidine tag which contribute 2.8 kDa to the size of *Mt*Bgl3a. The nominal mass discrepancy observed for *Mt*Bgl3a might be explained by the existence of Asn-Xaa-Ser/Thr sequons and Ser-Thr residues, which are known to be a prerequisite for *N*- and *O*-glycosylation post-translational modifications respectively. Indeed, 2 *N*-glycosylation and 95 potential *O*-glycosylation sites (50 Ser and 45 Thr) were predicted by using the NetNGlyc 1.0 server (http://www.cbs.dtu.dk/services/NetNGlyc/) and the NetOGlyc 3.1 server (http://www.cbs.dtu.dk/services/NetOGlyc/). The same observation was indicated in the heterologous expression of a feruloyl esterase from the same thermophilic fungus (StFaeB) in *P. pastoris*, where 3 potential *N*-glycosylation sites were predicted (Topakas et al., 2012). The calculated pI value of the translated recombinant *Mt*Bgl3a was found 4.0, which is close to the experimentally determined value range found from the IEF in the pH range of 3–9 (multiple bands in the range of 3.8–4.5; Fig. 2B). Glycosylation patterns may shift the pI as a result of the charged carbohydrate groups added to the molecule during post-translational modification. *P. pastoris* glycosylation patterns have been showed to vary considerably in the length and the type of oligosaccharides, thus resulting in diverse structural heterogeneity of the protein population. Within the same cell, two different molecules of the same protein may have different oligosaccharides, even they have been exposed to the same enzymes and glycosylation machinery. It has been observed that some proteins heterologously expressed in *P. pastoris* vary considerably in terms of the number of the mannose units added to the same polysaccharide core (Daly & Hearn, 2005). Both molecular mass and pI values of the isolated recombinant *Mt*Bgl3a are similar to other fungal β-glucosidases, such as β-glucosidase from *Penicillium brasilianum* (92.9 kDa, pI 3.9) (Krogh et al., 2010).

### Characteristics of *Mt*Bgl3a

The purified recombinant *Mt*Bgl3a was assayed for its activity toward different substrates under the standard assay conditions (Table 2). β-Glucosidase was preferentially active against *p*-β-NPG when compared to cellobiose. The highest activity was observed with 1 mM *p*-β-NPG (97.7 U/mg) followed by laminarin (52.0 U/mg), 10 mM cellobiose (30.7 U/mg) and lichenan (20.6 U/mg). The purified enzyme had lower activity on *p*-NPCell and barley-β-glucan, whereas no detectable activity towards *p*-α-NPGal, *p*-α-NPG, CMC, xylans (wheat and birchwood) and Avicel was observed. Although polymers are not usually substrates for β-glucosidases, *Mt*Bgl3a can hydrolyze long glucans, such as laminarin and lichenan and in this respect resembles an exoglucanase. Several β-glucosidases have been found to hydrolyze laminarin and/or lichenan (Mamma, Hatzinikolaou & Christakopoulos, 2004; Riou et al., 1998). According to their specific activity β-glucosidases are classified into three major groups including aryl β-glucosidases with a strong affinity for aryl β-glucosides, cellobiases, which only hydrolyze oligosaccharides (including cellobiose) and β-glucosidases that are active with both type of substrates (Enari & Niku-Paavola, 1987). The above results indicate that β-glucosidase *Mt*Bgl3a was active against both aryl β-glucosides and cellobiose therefore it can be concluded that belong to the last group.

**Table 2:** Activity of purified *Mt*Bgl3a on polysaccharide substrates. Activity was measured, as described in “Materials and methods” section. Activity not detected for substrates *p*-α-NPG, *p*-α-NPGal, wheat arabinoxylan, CMC, Avicel, filter paper and birchwood xylan. The experiment was carried out in triplicates.

Substrate	Specific activity
	(U/mg protein)
*p*-β-NPG (1 mM)	97.7 ± 1.07
*p*-β-NPGal (5 mM)	traces
*p*-NPCell (1 mM)	15.9 ± 0.07
cellobiose (10 mM)	30.7 ± 0.97
laminarin (0.5%)	52.0 ± 2.20
lichenan (1%)	20.6 ± 0.04
barley β-glucan (1%)	12.4 ± 0.18

**Figure 3:**
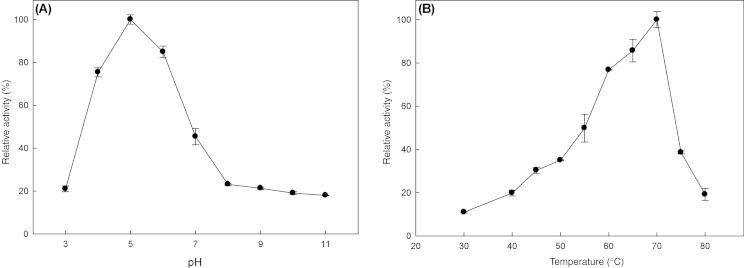
Effect of pH (A) and temperature (B) on the activity of *Mt*Bgl3a.

**Figure 4:**
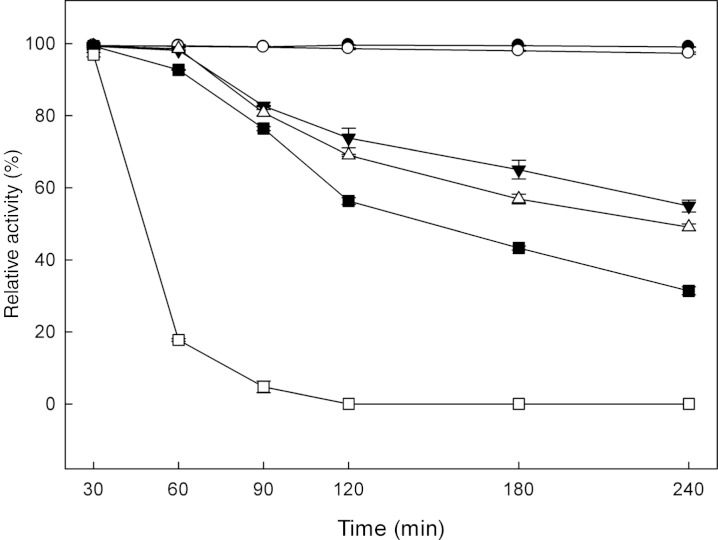
The thermal stability of *Mt*Bgl3a preincubated at different temperatures (pH 6.5, 30–65 °C) in the absence of substrate and assayed for residual activity on p-β-NPG under standard assay conditions. Incubation temperatures: 30 °C (●), 40 °C (○), 50 °C (▼), 55 °C (∆), 60 °C (■) and 65 °C (□).

The effect of pH and temperature on *Mt*Bgl3a enzymatic activity and stability was evaluated. The enzyme presented the highest activity levels at pH 5.0 and the >80% of the peak activity was displayed at pH 6, while the activity drops rapidly for pH less than 4 or higher than 7 (Fig. 3A). The enzyme was found remarkably stable in the pH range 3–7 after 24 h of incubation, retaining almost 90% of the initial activity (data not shown). The specific activity of the *Mt*Bgl3a at different temperatures under standard assay conditions was measured and the enzyme exhibited its optimal activity at 70 °C (Fig. 3B). The β-glucosidase was fairly stable up to 60 °C for 60 min and retained 56.3% of its activity after 2 h preincubation at the same temperature (Fig. 4). *Mt*Bgl3a exhibits half lives of 274 min, 214 min and 143 min at 50 °C, 55 °C and 60 °C, respectively. Other β-glucosidases showing similar values for optimal temperature and pH of enzymatic activity have been isolated from different fungal species, such as *A. fumigatus* (pH 6, 60 °C; Liu et al., 2012), *P. brasilianum* (pH 4.8, 70 °C; Krogh et al., 2010) and *T. koningii* (pH 5, 50 °C; Lin et al., 2010).

The effect of various chemical compounds and other substrates at 10 mM was tested on the activity of β-glucosidase (Table 3). The enzyme was slightly inhibited by Ca^2+^,Co^2+^, EDTA and SDS while, it was activated by Mn^2+^,Mg^2+^,Zn^2+^ and Cu^2+^. β-Glucosidases of microbial origin are usually affected by EDTA and the cations Mg^2+^,Ca^2+^,Co^2+^,Mn^2+^,Cu^2+^, and Zn^2+^ at 1.0 mM (Venturi et al., 2002; Peralta et al., 1997). It has been demonstrated that some fungal β-glucosidases are activated by several cations, including Ca^2+^,Mg^2+^,Co^2+^, and/or Mn^2+^ (Riou et al., 1998; Karnchanatat et al., 2007).

**Table 3:** Effect of metal ions and other chemical reagents (10 mM each) on β-glucosidase *Mt*Bgl3a. The experiment was carried out in triplicates.

Metal ion or reagent	Residual activity (%)
KCl	103%
ZnSO_4_	149%
CuSO_4_	208%
MgSO_4_	110%
MnSO_4_	167%
CoCl_2_	85%
CaCl_2_	94%
EDTA	90%
urea	95%
SDS	91%

### Determination of *Mt*Bgl3a kinetic parameters – inhibition studies

The Michaelis–Menten constants were determined for *p*-β-NPG and cellobiose. The recombinant β-glucosidase showed higher specificity for the hydrolysis of *p*-β-NPG compared to cellobiose, exhibiting *K*_*m*_ values of 0.39 ± 0.12 mM and 2.64 ± 0.30 mM, respectively. However, both substrates were hydrolysed by the enzyme exhibiting similar velocities of 47.9 ± 3.8 and 49.4 ± 2.4 µmol/min/mg for *p*-β-NPG and cellobiose, respectively. A broad range of *K*_*m*_ values for *p*-β-NPG and cellobiose has been reported for β-glucosidases produced from different fungal sources, such as *Aspergillus oryzae* (*K*_*m*_ = 0.29 mM for *p*-β-NPG; Langston, Sheehy & Xu, 2006), *Aspergillus niger* (*K*_*m*_ = 0.57 mM for *p*-β-NPG and 0.88 mM for cellobiose; Chauve et al., 2010) and *Trichoderma reesei* (*K*_*m*_ = 0.38 mM for *p*-β-NPG and 1.36 mM for cellobiose; Chauve et al., 2010).

The effect of different amounts of D-glucose (0–25 mM), D-xylose (0–10 mM) and D-gluconic acid (0–10 mM) on the hydrolysis of *p*-β-NPG by β-glucosidase was investigated (Table 4). The enzyme was competitively inhibited by D-glucose and D-xylose with *K*_*i*_ values of 282 µM and 30 µM, respectively. β-Glucosidase is frequently a rate-limiting factor during enzymatic hydrolysis of cellulose and is very sensitive to D-glucose inhibition (Mamma, Hatzinikolaou & Christakopoulos, 2004; Riou et al., 1998; Karnchanatat et al., 2007). Most of the microbial β-glucosidases, reported to date, are competitively inhibited by D-glucose and exhibit *K*_*i*_ values ranging from as low as 0.2 mM to no more than 100 mM (Pitson, Seviour & McDougall, 1997; Karnchanatat et al., 2007; Mamma, Hatzinikolaou & Christakopoulos, 2004; Seidle et al., 2004; Harhangi et al., 2002; Lin, Pillay & Singh, 1999; Ferreira Filho, 1996; Parry et al., 2001). However, several fungal β-glucosidases show high glucose tolerance with *K*_*i*_ values of more than 100 mM (Riou et al., 1998).

**Table 4:** Kinetic parameters of inhibition of *p*-β-NPG hydrolysis by 0–25 mM glucose, 0–10 mM D-xylose and 0–10 mM glucono-δ-lactone/gluconic acid. The *K*_*i*_ values given are the averages of separate experiments on four different substrate concentrations performed in duplicate.

Inhibitor (mM)	*K*_*m*_ (mM)	*V*_max_ (µmol/min/mg protein)	*K*_*i*_ (µM)
Glucose	7.262 ± 1.2	100.656 ± 38	282 ± 100
Xylose	9.017 ± 3.5	389.6 ± 72	30 ± 5
glucono-δ-lactone/gluconic acid	1.84 ± 0.5	205.4 ± 49	22 ± 2

D-Gluconic acid, as a transition state analogue, is by far the most potent inhibitor for the microbial β-glucosidases (Harhangi et al., 2002). Cellulolytic fungi have been proposed to own an oxidoreductive cellulolytic system composed by different enzymes, such as lytic polysaccharide monooxygenases members of GH family 61 proteins, that co-exists with the well-studied fungal cellulases resulting in efficient lignocellulose conversion (Dimarogona et al., 2012; Langston et al., 2011). These enzymes act through a mechanism that involves an hydrolytic and an oxidative step, thus generating two new chain ends on the crystalline surface, one normal non-reducing and an “oxidized reducing end”, i.e. an aldonic acid (Forsberg et al., 2011; Langston et al., 2011). Thus the use of β-glucosidases with tolerance for these oxidized derivatives, such as D-gluconic acid seems to be a promising approach. Like other fungal β-glucosidases, the *Mt*Bgl3a was competitively inhibited by D-gluconic acid (Pitson, Seviour & McDougall, 1997; Parry et al., 2001; Riou et al., 1998) with a *K*_*i*_ value of 22 µM. *K*_*i*_ values reported for D-gluconic acid are in the range of 3–30 µM (Harhangi et al., 2002; Pitson, Seviour & McDougall, 1997; Parry et al., 2001) with exception of the β-glucosidase from *A. oryzae*, which exhibited a *K*_*i*_ value of 12.5 mM (Riou et al., 1998).

### Effect of alcohols and transglycosylation activity

**Figure 5:**
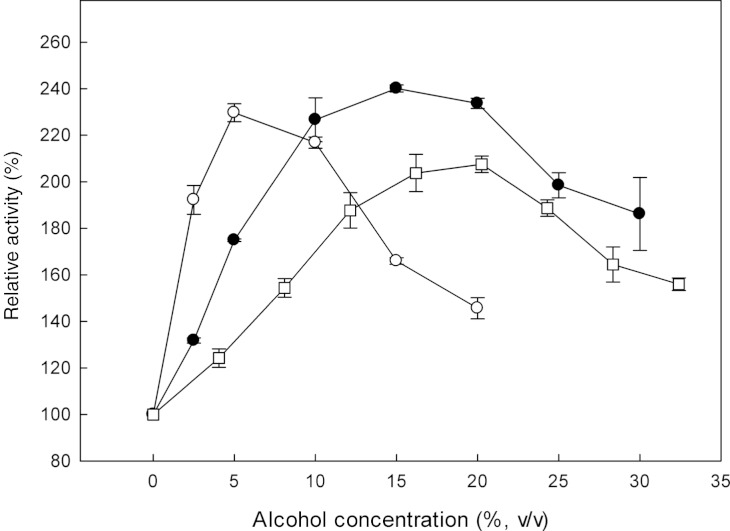
Effect of increasing concentrations of alcohols, such as methanol (□), ethanol (●) and propanol (○) on the activity of *Mt*Bgl3a.

β-Glucosidases catalyse the transglycosylation reaction in an aqueous solution, in the presence of a second nucleophile stronger than water, such as methanol or ethanol (Tsitsimpikou et al., 1997). The effect of alcohols on the specific activity of the recombinant β-glucosidase was studied, by determining the activity on *p*-β-NPG in the presence of 0–33% (v/v) methanol, 0–30% (v/v) ethanol and 0–20% (v/v) propanol (Fig. 5). In the presence of these alcohols, an increase in enzyme activity was observed. Analysis of the reaction products revealed that the optimum methanol concentration was 20% (v/v). At concentrations higher than 20% (v/v), the activation was decreased probably due to the denaturation effect of methanol, as many proteins break down in response to alcohol exposure. Furthermore, ethanol and propanol stimulated the activity of β-glucosidase to concentrations up to 15% (v/v) and 5% (v/v), respectively. These results indicate that the presence of short chain alcohols have a positive influence on the hydrolytic activity of β-glucosidase. It has been reported that the change in polarity of the medium induced by alcohols could stabilize enzyme conformation (Mateo & Di Stefano, 1997). Activation by short chain alcohols has been earlier observed for β-glucosidase from *Thermoascus aurantiacus* (Parry et al., 2001), *A. oryzae* (Riou et al., 1998), *Fusarium oxysporum* (Christakopoulos et al., 1994).

*Mt*Bgl3a remained stable after 6 h of incubation at different ethanol concentrations up to 50% (v/v) at 30 °C. This result is important at the SSF process for industrial ethanol production since the enzyme and ethanol coexist in the reactor. Various investigations have shown that ethanol reduces the enzyme activities of cellulaces (Wu & Lee, 1997; Jorgensen et al., 2007) thus the presence of an ethanol tolerant β-glucosidase appears to be critical for the efficiency of the process, as it will be able to remove cellobiose even at the late fermentation process eliminating its inhibitory effect.

**Figure 6:**
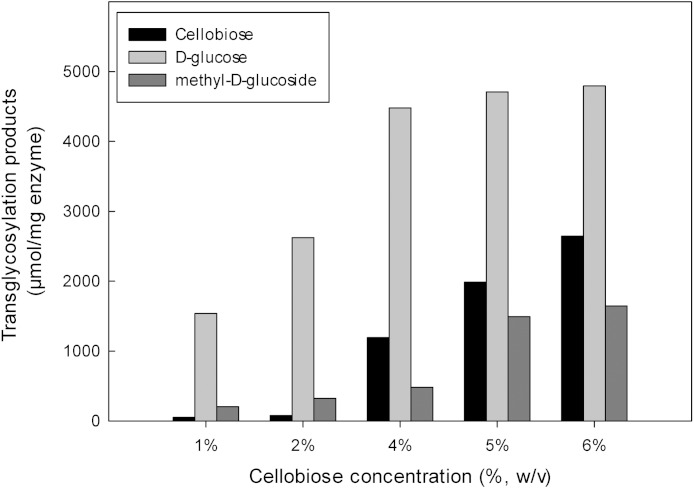
Effect of different amounts of cellobiose in transglycosylation reaction with methanol activity catalyzed by *Mt*Bgl3a.

The inductive effect of the methyl group and the transglycosylation activity of *Mt*Bgl3a were further investigated by studying the effect of increasing cellobiose concentration on the composition of the product mixture (Fig. 6). Methanol was used at the optimum concentration (20%, v/v) found previously for methyl-D-glucoside synthesis. Analysis of the reaction products by HPLC revealed that methyl-D-glucoside synthesis increased sharply at cellobiose concentrations above 4% (w/v) in the reaction. The *K*_*m*_ values of cellobiose for transglycosylation (methanolysis) were calculated to be 91 mM. Similar affinity towards cellobiose for transglycosylation (methanolysis) has been reported for β-glucosidase from *F. oxysporum* (*K*_*m*_ = 138.2 mM) (Tsitsimpikou et al., 1997). Methanol has a competitive role as a nucleophilic glycosyl acceptor, as glycosylation proceeds through a nucleophilic attack to the stronger nucleophilic character of methanol compared to that of water (Drueckhammer et al., 1991). Glycosidase-catalyzed transglycosylation is a promising alternative to classical chemical glycosylation methods, with numerous applications not only in the Food and Cosmetics but also in Pharmaceutical industry for the production of bioactive compounds (Pal et al., 2010). The transferase activity was also studied in β-glucosidase from *P. thermophila* (Yang et al., 2008), *Thermotoga neapolitana* (Park et al., 2005), thermophilic fungus *Melanocarpus sp.* (Kaur et al., 2007).

## Conclusion

Currently, heterologous expression is the main tool for the production of industrial enzymes, with *P. pastoris* being one of the favorite expression hosts for the heterologous expression of eukaryotic biocatalysts. In this study, the gene encoding *Mt*Bgl3a from *M. thermophila* was functionally expressed and secreted by the heterologous host *P. pastoris*. β-Glucosidases are of key importance, as they are needed to supplement the cellobiohydrolase and endoglucanase activities for ensuring final glucose release and at the same time decreasing the accumulation of cellobiose and shorter cellooligomers, which are known as product inhibitors for cellobiohydrolases. The low inhibition rate by glucose and ethanol renders this enzyme a good candidate for use in many biotechnological processes, including cellulose degradation, where combined stability is appreciated. In addition, the ability of the enzyme to catalyze transglycosylation reactions seems to be very promising for the synthesis of glycoside-containing compounds and bioactive products with potential application in both Food and Pharmaceutical industries.
